# Crystal Structure Manipulation of the Exchange Bias in an Antiferromagnetic Film

**DOI:** 10.1038/srep28397

**Published:** 2016-06-22

**Authors:** Wei Yuan, Tang Su, Qi Song, Wenyu Xing, Yangyang Chen, Tianyu Wang, Zhangyuan Zhang, Xiumei Ma, Peng Gao, Jing Shi, Wei Han

**Affiliations:** 1 International Center for Quantum Materials, Peking University, Beijing, 100871, P.R. China; 2 Collaborative Innovation Center of Quantum Matter, Beijing 100871, P.R. China; 3 Electron Microscopy Laboratory, School of Physics, Peking University, Beijing, 100871, P.R. China; 4 Department of Physics and Key Laboratory of Artificial Micro- and Nano-structures of Ministry of Education, Wuhan University, Wuhan 430072, P.R. China; 5 Department of Physics and Astronomy, University of California, Riverside, California 92521, USA

## Abstract

Exchange bias is one of the most extensively studied phenomena in magnetism, since it exerts a unidirectional anisotropy to a ferromagnet (FM) when coupled to an antiferromagnet (AFM) and the control of the exchange bias is therefore very important for technological applications, such as magnetic random access memory and giant magnetoresistance sensors. In this letter, we report the crystal structure manipulation of the exchange bias in epitaxial hcp Cr_2_O_3_ films. By epitaxially growing twined 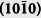 oriented Cr_2_O_3_ thin films, of which the *c* axis and spins of the Cr atoms lie in the film plane, we demonstrate that the exchange bias between Cr_2_O_3_ and an adjacent permalloy layer is tuned to in-plane from out-of-plane that has been observed in 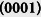 oriented Cr_2_O_3_ films. This is owing to the collinear exchange coupling between the spins of the Cr atoms and the adjacent FM layer. Such a highly anisotropic exchange bias phenomenon is not possible in polycrystalline films.

Exchange bias refers to the shift of the magnetic hysteresis loop of a ferromagnetic (FM) layer away from the zero magnetic field, resulting from the exchange interaction from an antiferromagnet (AFM) layer^1^,^2^,^3^,^4^. In practical applications such as magnetic random access memory and giant magnetoresistance sensors, etc.^5^,^6^, the exchange bias is used to “pin” the FM magnetization from switching in small magnetic fields so that the FM layer could serve as a fixed reference layer. In previous studies, it has been established that the exchange bias hinges on the spin orientations of the surface magnetic atoms in the AFM layer^7^,^8^,^9^,^10^,^11^,^12^,^13^. Since the direction of the spin orientations of the surface magnetic atoms is associated with the crystal structure of the AFM layer, the highly anisotropic exchange bias could be simply manipulated by the crystal orientation design. An ideal candidate AFM material to achieve this objective is the single crystalline Cr_2_O_3_ films with a hexagonal close packed (hcp) structure, of which the spin orientations of the Cr atoms are uniquely directed along the *c* axis and could dictate the direction of the exchange bias^7^,^14^,^15^.

In this letter, we report the manipulation of the exchange bias by controlling the surface spin configuration via crystal orientation design. By epitaxially growing 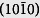 oriented Cr_2_O_3_ films on (001) oriented rutile TiO_2_ substrates, we force the *c* axis to lie in the film plane, which results in a strong in-plane exchange bias between the Cr_2_O_3_ film and an adjacent permalloy (Py) layer, whilst the perpendicular exchange bias is completely suppressed. The perpendicular exchange bias was previously shown in (0001) oriented Cr_2_O_3_ films^1^,^2^,^3^,^4^,^7^,^15^,^16^. Our results along with the previous studies demonstrate crystal structure manipulations of the exchange bias based on the collinear exchange coupling between the spins of the Cr atoms and the adjacent FM layer.

## Results and Discussion

**Figure 1:**
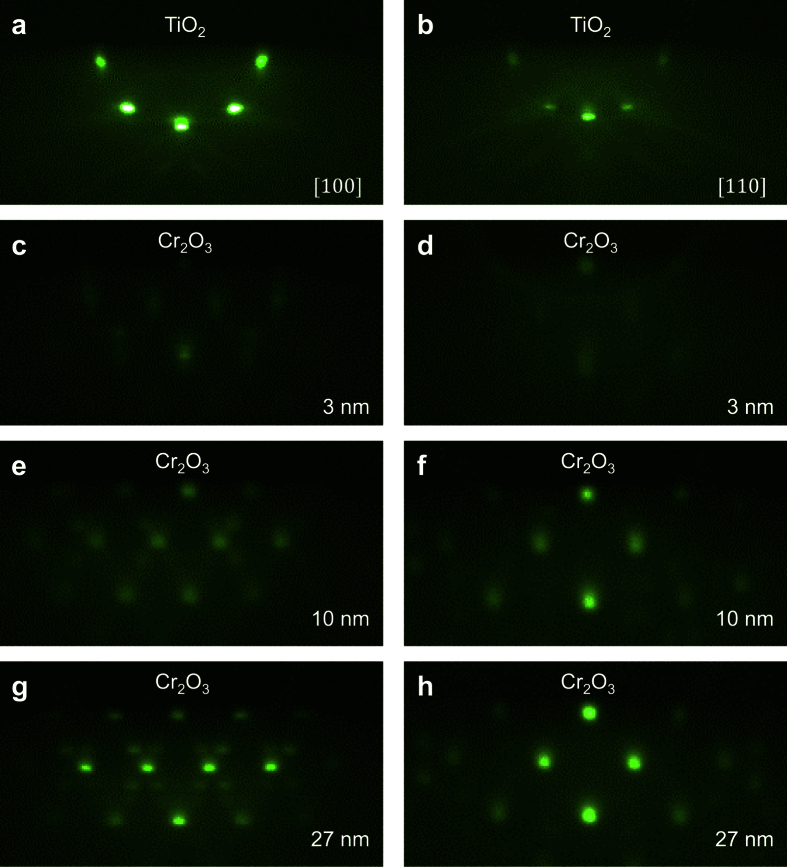
*In-situ* RHEED characterization for the 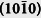 oriented Cr_2_O_3_ films grown on (001) oriented rutile TiO_2_ substrates. (**a**,**b**) The RHEED patterns of the rutile TiO_2_ substrate viewed from 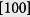 and 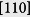 directions prior to the Cr_2_O_3_ growth. (**c–h**) The RHEED patterns of 3 nm (**c**,**d**), 10 nm (**e**,**f**), and 27 nm (**g**,**h**) Cr_2_O_3_ films, respectively. The figures in the left/right column are RHEED patterns viewed from TiO_2_ 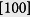/[110] direction.

The 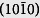 oriented Cr_2_O_3_ films are grown on the 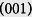 oriented rutile TiO_2_ substrates via laser molecular beam epitaxy (LMBE) with a base pressure of 2 × 10^−8^ mbar (see methods for details). Fig. 1a,b show the *in-situ* reflection high-energy electron diffraction (RHEED) characterization of the 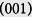 oriented TiO_2_ substrate’s surface viewed from the 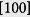 and 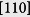 directions. After the initial growth of 3 nm Cr_2_O_3_, the RHEED pattern of the TiO_2_ substrate disappears and that of Cr_2_O_3_ starts to appear, as shown in Fig. 1c,d. As the thickness of Cr_2_O_3_ film increases, its RHEED pattern becomes brighter. Fig. 1e–h show the RHEED patterns of 10 nm and 27 nm Cr_2_O_3_ films, respectively. To be noted, four satellite RHEED spots are observed around each main diffraction spot viewed from the 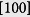 direction of the TiO_2_ substrates (Fig. 1e,g).

**Figure 2:**
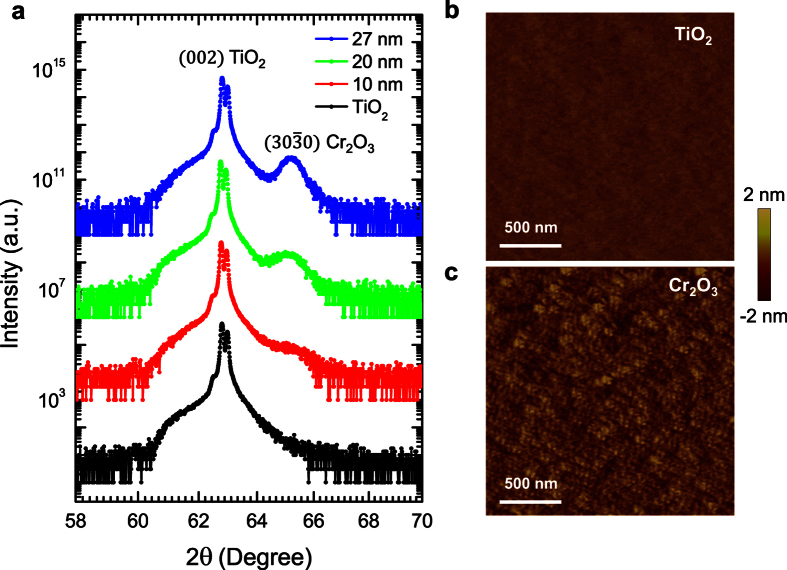
The crystalline structure and surface roughness of the 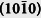 oriented Cr_2_O_3_ films. (**a**) X-ray diffraction measurement of the rutile TiO_2_ substrate and the 10 nm, 20 nm, and 27 nm Cr_2_O_3_ films, respectively. (**b**,**c**) AFM images of a typical TiO_2_ substrate and a 13 nm Cr_2_O_3_ film.

The crystalline structural properties of these Cr_2_O_3_ films are further characterized by x-ray diffraction (XRD). The θ–2θ scans of the rutile TiO_2_ substrate, 10 nm, 20 nm, and 27 nm Cr_2_O_3_ films are shown in Fig. 2a. The peak at 2θ of ~63 degrees corresponds to the 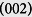 peak of the TiO_2_ substrates. For the 10 nm Cr_2_O_3_ film, a peak at 2θ of ~65 degrees is observed, which corresponds to the 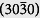 peak of the Cr_2_O_3_ crystal. As the thickness of Cr_2_O_3_ increases, the intensity of the peak at ~65 degrees becomes stronger. We note that for the 27 nm Cr_2_O_3_ film, only 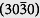 peak is detectable for the whole scan range (see supplementary information; Fig. S1), indicating good crystalline properties of the 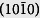 oriented Cr_2_O_3_ thin films. The surface morphology is characterized by atomic force microscopy (AFM). The root-mean-square (RMS) roughness is 0.09 nm for the rutile TiO_2_ substrate after annealing in the chamber (Fig. 2b). After the growth of 13 nm Cr_2_O_3_ film, the RMS roughness increases to 0.28 nm (Fig. 2c), indicating that the surface of the epitaxial Cr_2_O_3_ films is quite smooth.

**Figure 3:**
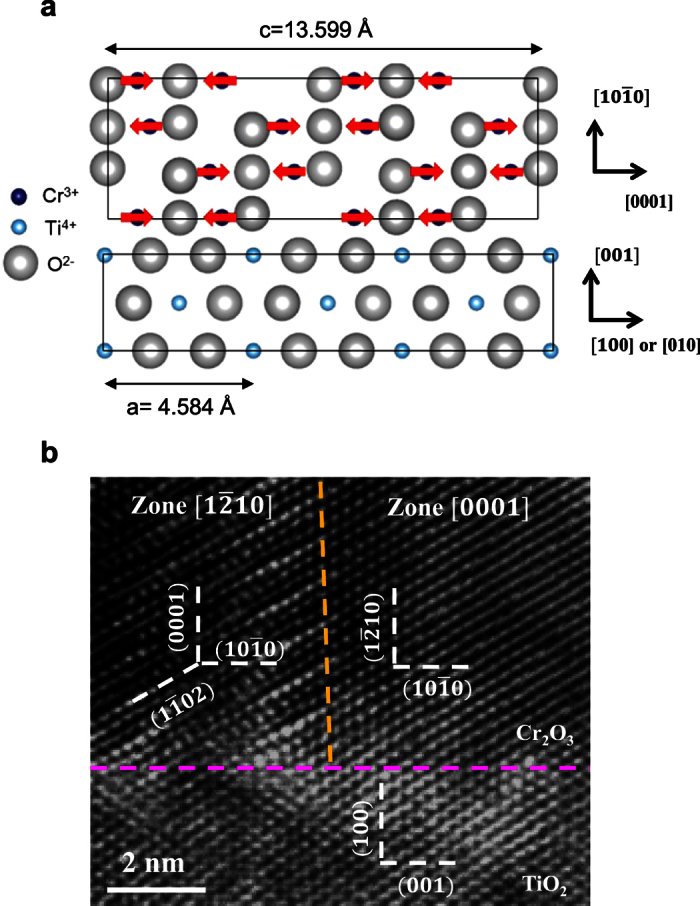
Crystalline structure of the 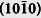 oriented Cr_2_O_3_ film by HRTEM. (**a**) The schematic drawing showing the atomic interface between Cr_2_O_3_ and TiO_2_. The spins of the Cr are parallel to the film plane, indicated by the red arrows. (**b**) HRTEM characterization. The pink dashed line indicates the interface between Cr_2_O_3_ and TiO_2_, and the yellow dashed line indicates the crystalline boundary between the zones 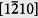 and 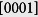 for the oriented Cr_2_O_3_ film. In the zone 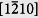, the [0001] directions of Cr_2_O_3_ is parallel to the 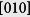, or 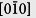
of the TiO_2_ substrate. Whilst in the zone [0001], the 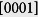 direction of Cr_2_O_3_ is parallel to the [100], or 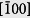 of the TiO_2_ substrate.

The epitaxial growth of the 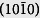 oriented Cr_2_O_3_ films on TiO_2_ is quite interesting, given the fact that Cr_2_O_3_ and TiO_2_ belong to totally different space groups. Cr_2_O_3_ has a hexagonal crystal structure, which belongs to the 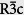 group^17^, while rutile TiO_2_ has a cubic structure, which belongs to the P4_2_ group^18^. However, the *c* lattice constant of Cr_2_O_3_ is 13.599 Å, and the *a* lattice constant of TiO_2_ is 4.584 Å, which results in a coincidental anion alignment with a lattice mismatch of only ~1.1% 
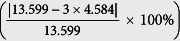
. Hence, the Cr_2_O_3_ films could be grown with the *c* axis lying in-plane and parallel to *a* or *b* axis of the TiO_2_ substrates (Fig. 3a). As the TiO_2_ crystal’s *ab* plane has four-fold symmetry, which could result in four-fold in-plane rotational symmetry of the crystalline structure of the Cr_2_O_3_ thin films. To investigate this, HRTEM is used to characterize the interfacial structure properties between Cr_2_O_3_ and TiO_2_ viewed from the 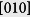 direction of the TiO_2_ substrate. As shown in Fig. 3b, a sharp interface with TiO_2_ is observed, as indicated by the pink dashed line. For Cr_2_O_3_, two distinct zones of the crystalline structures are observed, one of which is denoted as zone 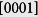, and the other one is denoted as zone 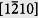. The boundary of these two zones is marked by a yellow dashed line. For zone [0001], the *c* axis of Cr_2_O_3_ is parallel to the 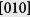 direction of the TiO_2_ substrate, and the six-fold symmetry pattern of the basal plane can be identified. For zone 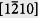, the *c* axis is parallel to the 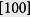 direction of the TiO_2_ substrate. The crystal orientation of Cr_2_O_3_ films has been shown to be highly associated to the substrate crystalline structures. For example, preferential 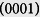 oriented growth of Cr_2_O_3_ is achieved on 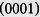 oriented Al_2_O_3_, Co, and 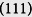 oriented Cu and 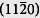 oriented Cr_2_O_3_ is grown on Fe 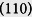 films^15^,^19^,^20^. The growth mode of 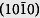 Cr_2_O_3_ film in our study is similar to that observed in an earlier report for the 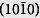 oriented Fe_2_O_3_ films grown on rutile 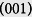.TiO_2_ substrates^21^.

**Figure 4:**
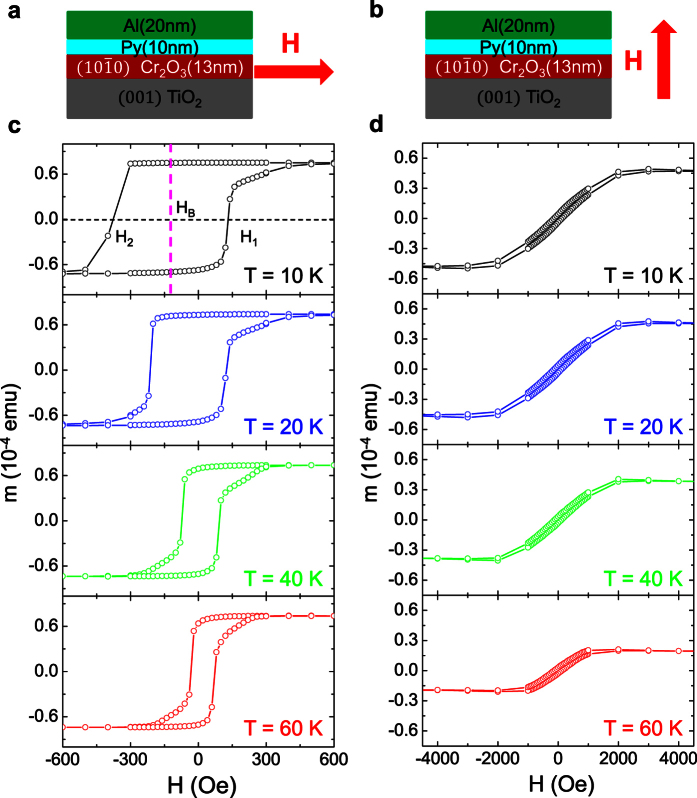
The characterization of the exchange bias for the sample of TiO_2_/Cr_2_O_3_ (13 nm)/Py (10 nm)/Al (20 nm). (**a**,**b**) Schematic drawings of the sample structure and the measurement geometry for in-plane exchange bias and perpendicular exchange bias, respectively. (**c**) The magnetization hysteresis loops measured as a function of the in-plane magnetic field along the TiO_2_ 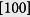 direction at 10 K, 20 K, 40 K, and 60 K, respectively. *H*_*1*_ and *H*_*2*_ indicate the coercive fields for the magnetization of Py and *H*_*B*_ indicates the exchange bias. (**d**) The magnetization curves measured as a function of the out-of-plane magnetic at 10 K, 20 K, 40 K, and 60 K, respectively.

Interestingly, with the *c* axis lying in the film plane, the spin orientations of the Cr atoms also lie in-plane in these 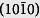 oriented Cr_2_O_3_ film, as schematically shown in Fig. 3a. This is very different from previously reported (0001) oriented Cr_2_O_3_ films grown on Al_2_O_3_ substrates, of which both the spin orientations and the exchanges bias are perpendicular to the films^7^,^15^,^16^. To study the exchange bias of the epitaxial Cr_2_O_3_ films (see Methods for details), we deposit 10 nm Py on top of the 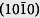 oriented Cr_2_O_3_ films and measure the magnetic hysteresis loops by Magnetic Properties Measurement System (MPMS; Quantum Design) with both in-plane and out-of-plane magnetic fields at various temperatures (schematic drawings shown in Fig. 4a,b). The in-plane magnetic hysteresis loop are first measured. Prior to the measurement, the sample is cooled from 400 to 10 K in an in-plane magnetic field of 1000 Oe along the [100] direction of the TiO_2_ substrate, which is much smaller than the spin-flop field of several Tesla for Cr_2_O_3_ reported previously^22^. By cooling through the blocking temperature, the magnetization direction of the Py sets the surface spin configurations of the Cr_2_O_3_ films. Then, we measure the magnetization of the Py as a function of the in-plane magnetic field along the [100] direction of the TiO_2_ substrate (Fig. 4a) from 10 to 300 K. After subtracting a linear background which is mainly due to the diamagnetic response of the rutile TiO_2_ substrate, the shifted magnetic hysteresis loops of Py are displayed in Fig. 4c. At 10 K, as the magnetic field ramps from negative to positive, a sharp jump in magnetic moment occurs at ~110 Oe, but the jump occurs at ~ −400 Oe on the return sweep. These two switching fields are labeled as *H*_*1*_ and *H*_*2*_, respectively, as indicated in the top panel of the Fig. 4c. The exchange bias field (*H*_*B*_) is defined by the mean value of the *H*_*1*_ and *H*_*2*_, i.e. 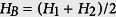. As the temperature increases, the exchange bias field steadily decreases from the low temperature value.

To characterize the anisotropy of the exchange bias effect, magnetic hysteresis loops are measured with a magnetic field perpendicular to the films (Fig. 4b). The same field cooling procedure as with the in-plane magnetic fields is adopted. The out-of-plane magnetization curves measured at 10, 20, 40 and 60 K are shown in Fig. 4d. The symmetric magnetization hysteresis loops indicate a negligible perpendicular exchange, which is in stark contrast to the out-of-plane exchange bias observed in the 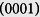 oriented Cr_2_O_3_ films. The highly anisotropic exchange bias phenomenon can be attributed to the crystalline orientation difference of the Cr_2_O_3_ films. In hcp structures, the c-axis direction dictates the spin orientations of the magnetic atoms. In the 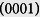 oriented Cr_2_O_3_ films, the spin orientations of the Cr atoms are perpendicular to the films, whereas in the 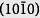 oriented Cr_2_O_3_ films, the spins of the Cr atoms lie in the film plane. These results further indicate the direct collinear exchange coupling between the spins of the Cr atoms and the adjacent Py layer.

**Figure 5:**
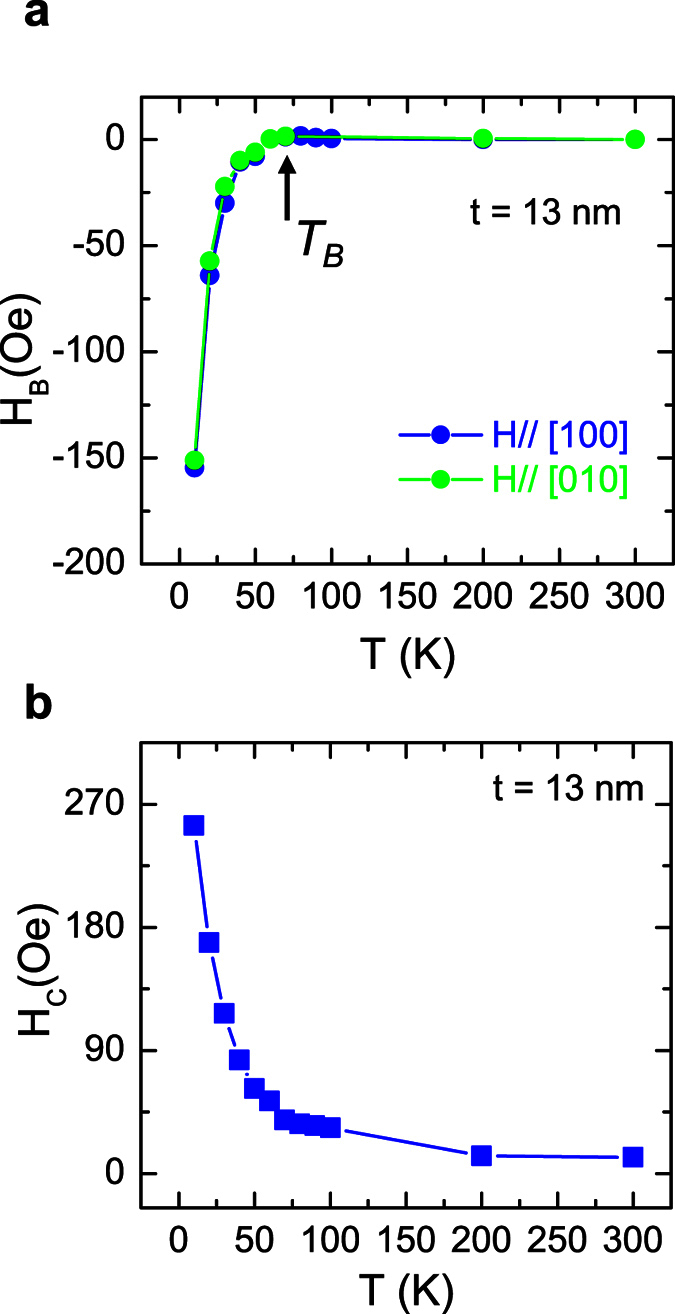
The exchange bias and the coercive field as a function of temperature for the sample TiO_2_/Cr_2_O_3_ (13 nm)/Py (10 nm)/Al (20 nm). (**a**) The exchange bias field as a function of the temperature for magnetic field along the TiO_2_ 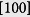, and 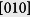 directions, respectively. *T*_*B*_ indicates the blocking temperature, above which the exchange bias becomes zero. (**b**) The coercive field of the Py as a function of the temperature.

Fig. 5a,b summarize the in-plane exchange bias field and in-plane conceive field (*H*_*C*_), where 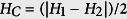, for the sample consisting of the 13 nm Cr_2_O_3_ films and 10 nm Py as a function of the temperature. As the temperature increases from 10 to 60 K, the in-plane exchange bias field (Fig. 5a, Blue dots) decreases quickly from ~ −150 Oe to almost 0 Oe, where the sign depends on the magnetic field direction during magnetic cooling. No exchange bias is observable at and above 60 K, which implies a blocking temperature (*T*_*B*_) of ~60 K. An abrupt increase in *Hc* below 60 K is another property of exchange biased Py, which is due to the formation of the AFM order in this 13 nm Cr_2_O_3_ thin film^2^. As there are two crystalline zones of 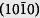 oriented Cr_2_O_3_, as indicated in zones 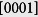 and 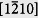, we also measure the exchange bias in the direction along the TiO_2_ 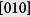 direction. Almost identical exchange biases are observed at each temperature (Fig. 5a, Green dots).

**Figure 6:**
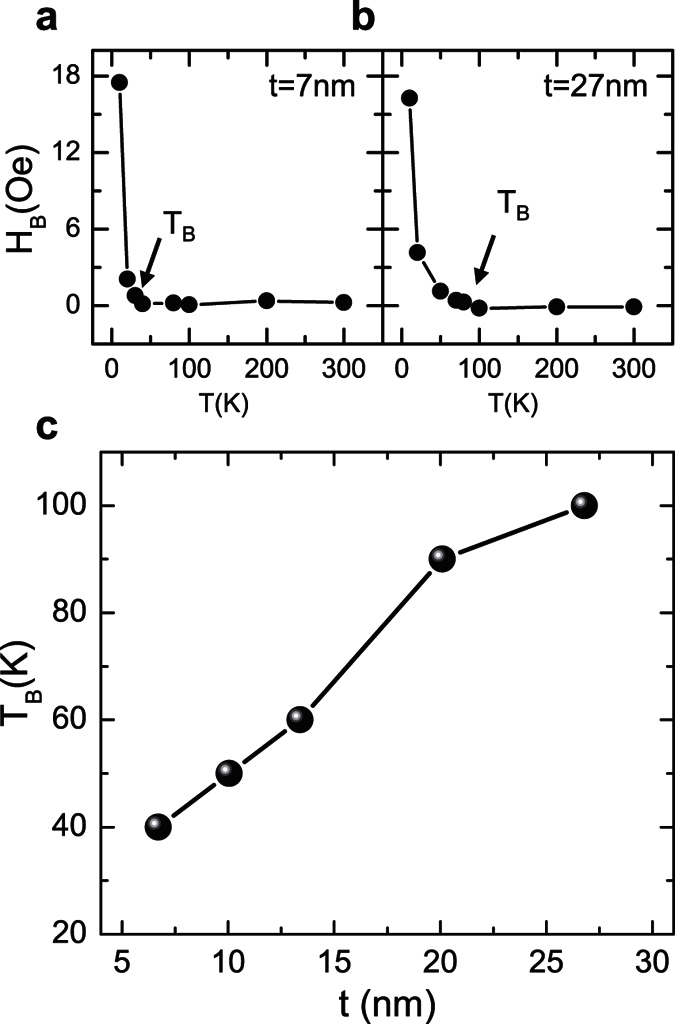
The exchange bias and blocking temperatures for the Cr_2_O_3_ films of various thicknesses. (**a**,**b**) The exchange bias as a function of the temperature for 7 nm and 27 nm Cr_2_O_3_ films, respectively. (**c**) The blocking temperature as a function of the Cr_2_O_3_ film thicknesses (*t*) for the samples TiO_2_/Cr_2_O_3_ (*t*)/Py (10 nm)/Al (20 nm).

The measured *T*_*B*_ of 13 nm Cr_2_O_3_ is ~60 K, which is much lower compared to the value reported on (0001) oriented bulk Cr_2_O_3_ single crystals^15^. In antiferromagnetic films, it has been known that *T*_*B*_ is highly related to Neel temperature (*T*_*N*_), and is usually slightly lower than the *T*_*N*_. Both *T*_*B*_ and *T*_*N*_ increase as the AFM thickness increases due to finite-size effects^2^,^12^,^23^,^24^. To obtain the *T*_*B*_ as a function of the thicknesses of the Cr_2_O_3_ thin films, the in-plane exchange bias for the samples consisting of 7, 10, 20 and 27 nm Cr_2_O_3_ films and 10 nm Py bilayer films are also measured. Fig. 6a,b show the exchange bias as a function of temperature for the bilayer structures consisting of Cr_2_O_3_ (7 nm)/Py (10 nm) and Cr_2_O_3_ (27 nm)/Py (10 nm), respectively. The blocking temperatures of these two structures are determined to be 40 K and 100 K. The blocking temperature increases as the thickness of the Cr_2_O_3_ films increases, as shown in Fig. 6c. For the 27 nm Cr_2_O_3_ film, the blocking temperature is only ~100 K, which is far below the T_B_ of bulk Cr_2_O_3_. One possible reason is the non-trivial finite size effects arising from the grain boundaries or oxygen defects in the Cr_2_O_3_^25^.

## Conclusion

In summary, we have demonstrated the manipulation of the exchange bias of the Cr_2_O_3_ thin films by controlling the surface spin orientations of the Cr atoms via crystal orientation design. For the epitaxial growth of 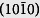 oriented Cr_2_O_3_ films, the spin configurations of Cr atoms give rise to only in-plane exchange bias at the interface between Py and the Cr_2_O_3_ thin films, while no perpendicular exchange bias is observed. Our results along with previous studies on (0001) oriented Cr_2_O_3_ films indicate the collinear exchange coupling between the spins of the Cr atoms and the adjacent FM layer.

## Methods

### Cr_2_O_3_ films growth

The 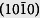 oriented Cr_2_O_3_ films are grown on the (001) oriented rutile TiO_2_ substrates via laser molecular beam epitaxy (LMBE) with a base pressure of 2 × 10^−8^ mbar. Prior to the Cr_2_O_3_ growth, the substrate temperature is increased to 350 °C with a rate of 20 °C/min in the chamber with an oxygen partial pressure of 0.08 mbar. Then the Cr_2_O_3_ film is deposited from a Cr_2_O_3_ target with a laser power of (8.0 ± 0.2) mJ and a frequency of 2.0 Hz. The thickness of the Cr_2_O_3_ thin film (*t*) is determined from the cross section high resolution transmission electron microscopy.

### Exchange bias measurement

A 10 nm Py is grown on top of the 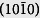 oriented Cr_2_O_3_ films by RF magnetron sputtering with a growth rate of 0.02 Å/s. A capping layer of 20 nm aluminum is deposited prior to taking the samples out of this sputtering chamber to prevent oxidization of Py. Magnetic Properties Measurement System (MPMS; Quantum Design) is used to measure the magnetic hysteresis loops to determine the exchange bias.

## Additional Information

**How to cite this article**: Yuan, W. *et al*. Crystal Structure Manipulation of the Exchange Bias in an Antiferromagnetic Film. *Sci. Rep.* **6**, 28397; doi: 10.1038/srep28397 (2016).

## Supplementary Material

Supplementary Information

## References

[b1] Meiklejohn W. H. & Bean C. P. New Magnetic Anisotropy. Phys. Rev. 105, 904–913 (1957).

[b2] Nogués J. & Schuller I. K. Exchange bias. J. Magn. Magn. Mater. 192, 203–232 (1999).

[b3] Kiwi M. Exchange bias theory. J. Magn. Magn. Mater. 234, 584–595 (2001).

[b4] Radu F. & Zabel H. Exchange Bias Effect of Ferro-/Antiferromagnetic Heterostructures . 97–184 (Springer Berlin Heidelberg, 2008).

[b5] Parkin S. S. P. . Exchange-biased magnetic tunnel junctions and application to nonvolatile magnetic random access memory (invited). J. Appl. Phys. 85, 5828–5833 (1999).

[b6] Wolf S. A. . Spintronics: A Spin-Based Electronics Vision for the Future. Science 294, 1488 (2001). 11711666 10.1126/science.1065389

[b7] Shiratsuchi Y., Nakatani T., Kawahara S.-i. & Nakatani R. Magnetic coupling at interface of ultrathin Co film and antiferromagnetic Cr_2_O_3_(0001) film. J. Appl. Phys. 106, 033903 (2009).

[b8] Nogués J., Lederman D., Moran T. J., Schuller I. K. & Rao K. V. Large exchange bias and its connection to interface structure in FeF2–Fe bilayers. Appl. Phys. Lett. 68, 3186–3188 (1996). 10.1103/PhysRevLett.76.4624 10061338

[b9] van der Zaag P. J., Ball A. R., Feiner L. F., Wolf R. M. & van der Heijden P. A. A. Exchange biasing in MBE grown Fe_3_O_4_/CoO bilayers: The antiferromagnetic layer thickness dependence. J. Appl. Phys. 79, 5103–5105 (1996).

[b10] Berkowitz A. E. & Takano K. Exchange anisotropy — a review. J. Magn. Magn. Mater. 200, 552–570 (1999).

[b11] Morales R. . Exchange-Bias Phenomenon: The Role of the Ferromagnetic Spin Structure. Phys. Rev. Lett. 114, 097202 (2015). 25793846 10.1103/PhysRevLett.114.097202

[b12] Wu J. . Direct Measurement of Rotatable and Frozen CoO Spins in Exchange Bias System of CoO/Fe/Ag (001). Phys. Rev. Lett. 104, 217204 (2010). 20867133 10.1103/PhysRevLett.104.217204

[b13] Nogués J., Moran T. J., Lederman D., Schuller I. K. & Rao K. V. Role of interfacial structure on exchange-biased FeF2-Fe. Phys. Rev. B 59, 6984–6993 (1999).

[b14] McGuire T. R., Scott E. J. & Grannis F. H. Antiferromagnetism in a Cr_2_O_3_ Crystal. Phys. Rev . 102, 1000–1003 (1956).

[b15] He X. . Robust isothermal electric control of exchange bias at room temperature. Nat. Mater. 9, 579–585 (2010). 20562879 10.1038/nmat2785

[b16] Shiratsuchi Y. . Detection and *In Situ* Switching of Unreversed Interfacial Antiferromagnetic Spins in a Perpendicular-Exchange-Biased System. Phys. Rev. Lett. 109, 077202 (2012). 23006398 10.1103/PhysRevLett.109.077202

[b17] Newnham Ε E. & Haan Υ. M. D. E. In Zeitschrift für Kristallographie - Crystalline Materials Vol. 117, 235 (1962).

[b18] Swope R. J., Smyth J. R. & Larson A. C. H in rutile-type compounds; I, Single-crystal neutron and X-ray diffraction study of H in rutile. American Mineralogist 80, 448–453 (1995).

[b19] Chen X. . Ultrathin chromia films grown with preferential texture on metallic, semimetallic and insulating substrates. Mater. Chem. Phys. 149–150, 113–123 (2015).

[b20] Sahoo S., Mukherjee T., Belashchenko K. D. & Binek C. Isothermal low-field tuning of exchange bias in epitaxial Fe ∕ Cr_2_O_3_∕Fe. Appl. Phys. Lett. 91, 172506 (2007).

[b21] Williams J. R., Wang C. & Chambers S. A. Heteroepitaxial growth and structural analysis of epitaxial α–Fe_2_O_3_(1010) on TiO_2_(001). J. Mater. Res. 20, 1250–1256 (2005).

[b22] Seki S. . Thermal generation of spin current in an antiferromagnet. Phys. Rev. Lett. 115, 266601 (2015). 26765011 10.1103/PhysRevLett.115.266601

[b23] Ambrose T. & Chien C. L. Finite-Size Effects and Uncompensated Magnetization in Thin Antiferromagnetic CoO Layers. Phys. Rev. Lett. 76, 1743–1746 (1996). 10060506 10.1103/PhysRevLett.76.1743

[b24] Imakita K.-i., Tsunoda M. & Takahashi M. Thickness dependence of exchange anisotropy of polycrystalline Mn3Ir/Co-Fe bilayers. J. Appl. Phys. 97, 10K106 (2005).

[b25] He X., Echtenkamp W. & Binek C. Scaling of the Magnetoelectric Effect in Chromia Thin Films. Ferroelectrics 426, 81–89 (2012).

