# Supplementary material for: Crystal Structure Manipulation of the Exchange Bias in an Antiferromagnetic Film
Source: Sci Rep. 2016 Jun 22;6:28397. doi: 10.1038/srep28397 (PMC4916595; doi:10.1038/srep28397)
Supplement: Supplementary Information [file srep28397-s1.pdf]

Supplementary Information for:

Crystal Structure Manipulation of the Exchange Bias in an Antiferromagnetic Film

Wei Yuan [1,2], Tang Su[1,2], Qi Song[1,2], Wenyu Xing[1,2], Yangyang Chen[1,2], Tianyu Wang[1,2], Zhangyuan Zhang[3,4], Xiumei Ma[3], Peng Gao[2,3], Jing Shi[5*], and Wei Han[1,2*]

[1]International Center for Quantum Materials, Peking University, Beijing, 100871, P. R. China

[2]Collaborative Innovation Center of Quantum Matter, Beijing 100871, P. R. China

[3]Electron Microscopy Laboratory, School of Physics, Peking University, Beijing, 100871, P. R. China

[4]Department of Physics and Key Laboratory of Artificial Micro- and Nano-structures of Ministry of Education, Wuhan University, Wuhan 430072, P. R. China

[5]Department of Physics and Astronomy, University of California, Riverside, California 92521, USA

*Correspondence to be addressed to: jing.shi@ucr.edu (J.S.) and weihan@pku.edu.cn (W.H.)

Figure S1

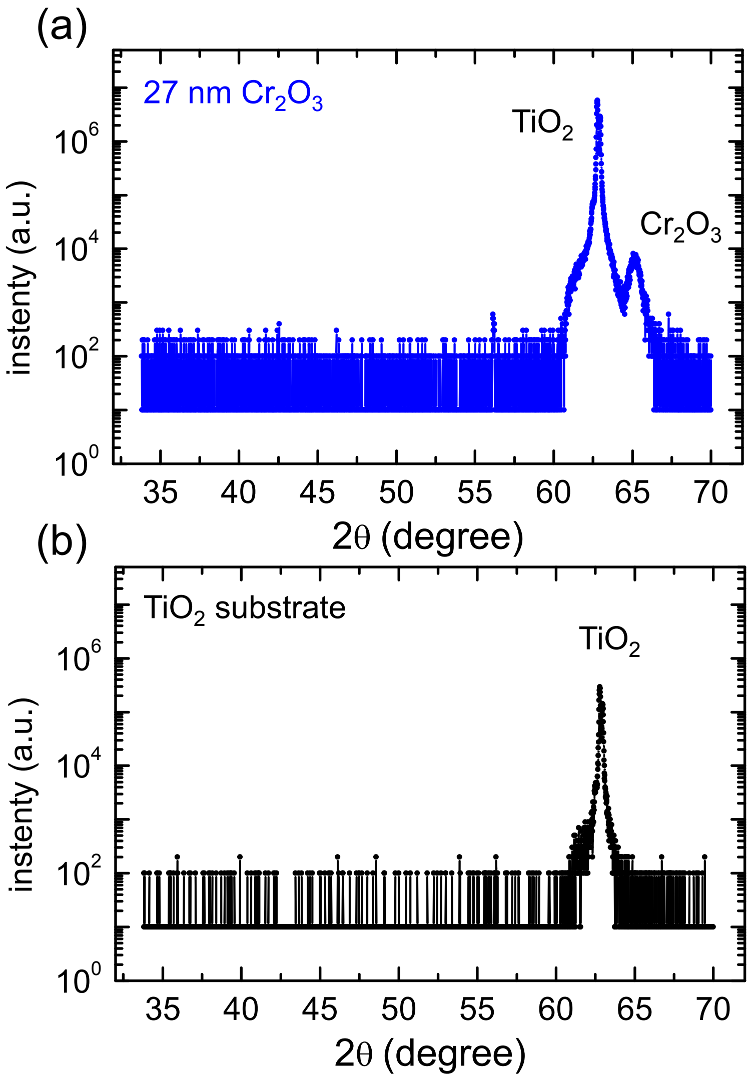

Fig. S1. The theta-2theta X-ray diffraction scans for 27 nm Cr_2O_3 films (a) and the rutile TiO_2 substrate (b), respectively.
